# Recombinant Peptide Production Platform Coupled with Site-Specific Albumin Conjugation Enables a Convenient Production of Long-Acting Therapeutic Peptide

**DOI:** 10.3390/pharmaceutics12040364

**Published:** 2020-04-16

**Authors:** Mijeong Bak, Junyong Park, Kiyoon Min, Jinhwan Cho, Jihyoun Seong, Young S. Hahn, Giyoong Tae, Inchan Kwon

**Affiliations:** 1 School of Materials Science and Engineering, Gwangju Institute of Science and Technology (GIST), Gwangju 61005, Korea; al4527@gm.gist.ac.kr (M.B.); kymin324@gist.ac.kr (K.M.); kroea2002@gist.ac.kr (J.C.); luvhhy@hanmail.net (J.S.); gytae@gist.ac.kr (G.T.); 2 Department of Biomedical Science and Engineering, Gwangju Institute of Science and Technology (GIST), Gwangju 61005, Korea; happydragon@gist.ac.kr; 3 Department of Microbiology, Immunology, and Cancer Biology, University of Virginia, Charlottesville, VA 22908, USA; ysh5e@virginia.edu

**Keywords:** recombinant therapeutic peptide, serum half-life extension, albumin conjugation

## Abstract

The number of therapeutic peptides for human treatment is growing rapidly. However, their development faces two major issues: the poor yield of large peptides from conventional solid-phase synthesis, and the intrinsically short serum half-life of peptides. To address these issues, we investigated a platform for the production of a recombinant therapeutic peptide with an extended serum half-life involving the site-specific conjugation of human serum albumin (HSA). HSA has an exceptionally long serum half-life and can be used to extend the serum half-lives of therapeutic proteins and peptides. We used glucagon-like-peptide 1 (GLP-1) as a model peptide in the present study. A “clickable” non-natural amino acid—p-azido-l-phenylalanine (AzF)—was incorporated into three specific sites (V16, Y19, and F28) of a GLP-1 variant, followed by conjugation with HSA through strain-promoted azide–alkyne cycloaddition. All three HSA-conjugated GLP-1 variants (GLP1_16HSA, GLP1_19HSA, and GLP1_28HSA) exhibited comparable serum half-lives *in vivo*. However, the three GLP1_HSA variants had different in vitro biological activities and in vivo glucose-lowering effects, demonstrating the importance of site-specific HSA conjugation. The platform described herein could be used to develop other therapeutic peptides with extended serum half-lives.

## 1. Introduction

**Figure 1:**
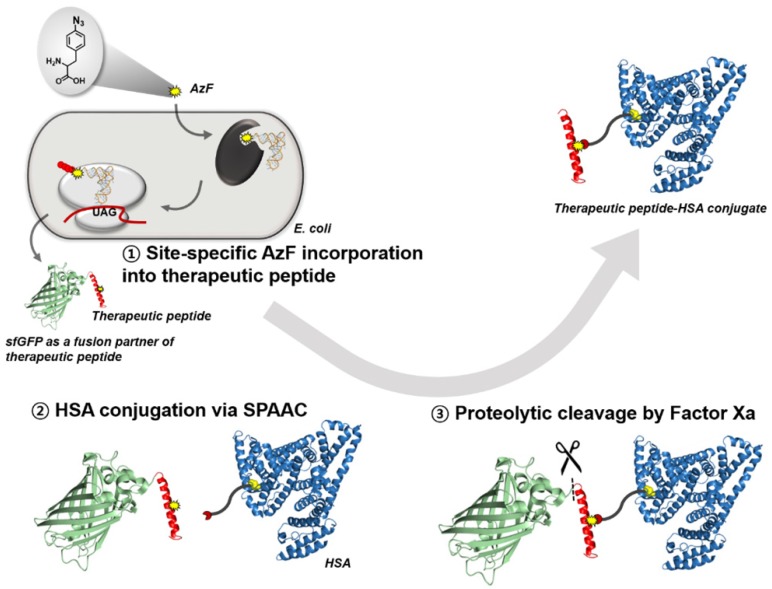
Schematic illustration of recombinant therapeutic peptide production platform coupled with site-specific conjugation of human serum albumin (HSA). ① A specific non-natural amino acid-incorporated therapeutic peptide with a fusion tag is overexpressed in Escherichia coli capable of “amber” stop codon suppression. ② Therapeutic peptide and HSA conjugation through biorthogonal reaction. ③ Cleavage of fusion tag using Factor Xa, which recognizes a specific cleavage site between the fusion tag and the recombinant therapeutic peptide.

Insulin was first isolated in the 1920s and was approved as a therapeutic peptide by the United States Food and Drug Administration (FDA) in 1982 [1]. Since then, the number of therapeutic peptides has increased rapidly, and their influence in the drug market has expanded. Sales of therapeutic peptides was expected to exceed 70 billion dollars in 2019, and numerous peptides are also being developed for clinical applications [2]. However, there are two major obstacles to the development of new therapeutic peptides: (1) the poor yield of large peptides (>30 amino acids) from conventional solid-phase synthesis [3,4], and (2) the intrinsically short serum half-life of peptides. To overcome these issues, in the present study, we investigated a recombinant peptide production platform coupled with site-specific conjugation of human serum albumin (HSA) to extend the serum half-life of a peptide (Figure 1).

Solid-phase synthesis has been widely used to produce small therapeutic peptides. However, its usefulness with regard to the production of high-molecular-weight therapeutic peptides (>30 amino acids) is limited because the overall synthesis yield is low, complicated isolation processes are required to remove impurities, and scale-up is difficult [3,4]. Alternatively, recombinant protein techniques have been used to biosynthesize peptides in host cells because the production yields of high-molecular-weight peptides are high, the isolation of target peptides is straightforward, and scale-up is convenient [5,6,7]. As most peptides alone are poorly expressed in host cells, a target peptide is often fused to a fusion protein partner, such as maltose-binding protein or glutathione S-transferase [8]. As a fusion partner, we chose superfolder green fluorescent protein (sfGFP) (Figure 1). sfGFP has several desirable features including high solubility and ready expression in Escherichia coli, and is therefore useful as a fusion partner [9,10]. After the expression and purification of the recombinant peptide fused with the fusion partner, the fusion partner should be removed to obtain only the target peptide. A specific sequence recognized by a protease—such as Factor Xa [11,12], thrombin [13,14], or tobacco etch virus (TEV) protease [15,16,17]—must be inserted into the fusion linker. In the present study, we chose the very popular protease Factor Xa to remove sfGFP from the fusion protein (Figure 1). sfGFP is highly resistant to most proteases [18], and can be used to avoid the generation of unwanted protein fragments during treatment with Factor Xa. Thereby we selected sfGFP as a fusion partner.

**Figure 2:**
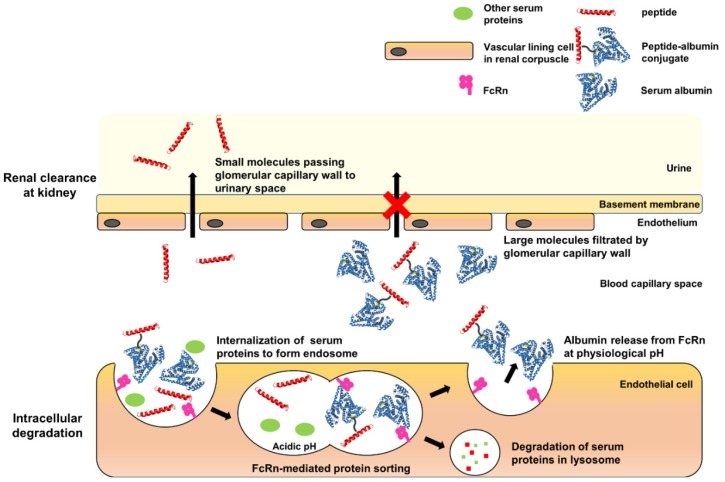
Schematic illustration of the effect of the half-life extension of the peptide drug by site-specific conjugation of human serum albumin (HSA). Conjugation with HSA results in an increase in the total molecular weight of the therapeutic peptide. In the glomerulus of the kidney, the therapeutic peptide (MW < 50 kDa) easily passes the basement membrane, moves into the urinary space, and is excreted from the body. However, the HSA-conjugated therapeutic peptide is filtered, resulting in reduced excretion in the urine. In the tissues, once the extracellular proteins have been internalized in the cells, HSA binds to FcRn, protein sorting occurs, and the peptide evades cellular degradation in the lysosomes. Finally, HSA and the HSA-conjugated therapeutic protein are released from FcRn (called the FcRn recycling mechanism).

Therapeutic peptides have an inherent drawback, i.e., a short serum half-life (several minutes to hours) [19], owing to rapid elimination from the circulation through several mechanisms, such as renal filtration, intracellular degradation, and proteolysis (Figure 2) [20]. The conventional method for extending the serum half-life of a therapeutic peptide is conjugation with polyethylene glycol (PEG) [21], which increases the apparent size of the peptide, making it more difficult for the kidneys to filter it. However, several concerns about PEG molecules have been raised recently, including their immunogenicity, accumulation in the body owing to poor degradability, and the loss of activity of therapeutic peptide upon conjugation [22,23]. Therefore, alternative strategies for extending the serum half-life of therapeutic peptides are required.

As alternatives to PEGylation, several strategies were developed [19,24]. First, in order to increase hydrodynamic volumes, therapeutic peptides/proteins were fused to recombinant PEG mimetics such as XTEN and PAS sequences or conjugated to carbohydrates such as hydroxyethyl starch and polysialic acid. Second, in order to evade intracellular degradation via neonatal Fc receptor (FcRn)-mediated recycling, Fc-domain or albumin was fused/conjugated to therapeutic peptides/proteins. Besides, conjugation of fatty acid, an albumin ligand, was also an effective way to extend the serum half-life of therapeutic peptides. In particular, HSA has a very long half-life (up to 3 weeks) in the human body and so has been investigated as a promising serum half-life extender of therapeutic peptides and proteins (Figure 2) [25,26,27,28]. Moreover, HSA has other desirable features such as low immunogenicity, excellent biocompatibility, and good degradability [28]. Therefore, it has been investigated as a candidate for extending the serum half-life of target peptides/proteins through either direct conjugation to the peptide/protein or indirect binding through ligands conjugated with the peptide/protein. Indirect binding to albumin using albumin-binding ligands (i.e., fatty acids and albumin-binding peptides) has drawbacks such as weak binding to the albumin and the potential immunogenicity of albumin ligands of non-human origin. The direct conjugation of albumin to the therapeutic peptide is often achieved by fusing albumin to the N- or C-terminus of the peptide, or by chemical conjugation. When a therapeutic peptide functions as a hormone or neurotransmitter, the albumin conjugated/fused to the N- or C-terminus of the peptide often hinders the interaction between the peptide and its receptor, resulting in reduced efficacy [29,30,31]. In particular, albumin conjugation to C-terminus of GLP-1 led to a 3300-fold reduction in its therapeutic efficacy [32]. Furthermore, the random conjugation of albumin to multiple sites on a therapeutic peptide results in a heterogeneous mixture of conjugates, requiring complicated purification processes. The site of albumin conjugation can be intentionally chosen on the peptide to avoid this problem. Recent advances in recombinant protein techniques allow site-specific modification of proteins/peptides. Site-specific incorporation of a non-natural amino acid into a target protein inside host cells has been achieved by the co-expression of an orthogonal pair comprising aminoacyl-tRNA synthetase/tRNA that is specific for the non-natural amino acid (Figure 1) [33,34]. Some non-natural amino acids in particular have “clickable” functional groups [35]. The site-specific incorporation of a clickable non-natural amino acid combined with the corresponding click chemistry enables site-specific conjugation to a target protein [36,37]. The site-specific conjugation of HSA to recombinant urate oxidase (a therapeutic protein for the treatment of hyperuricemia) and superfolder green fluorescent protein via strain-promoted azide–alkyne cycloaddition (SPAAC) effectively extends the serum half-life of the therapeutic protein [37,38]. Even for therapeutic peptides, SPAAC is preferable for albumin conjugation because it does not require a toxic catalyst and proceeds efficiently under mild conditions [39,40]. To conjugate HSA to the therapeutic peptide via SPAAC, we site-specifically introduced p-azido-l-phenylalanine (AzF) into the peptide using an orthogonal pair comprising Methanococcus jannaschii tyrosyl-tRNA synthetase/tRNA “amber” stop codon suppressor (MjTyrRS/MjtRNACUA), which is specific for AzF [41,42].

**Figure 3:**
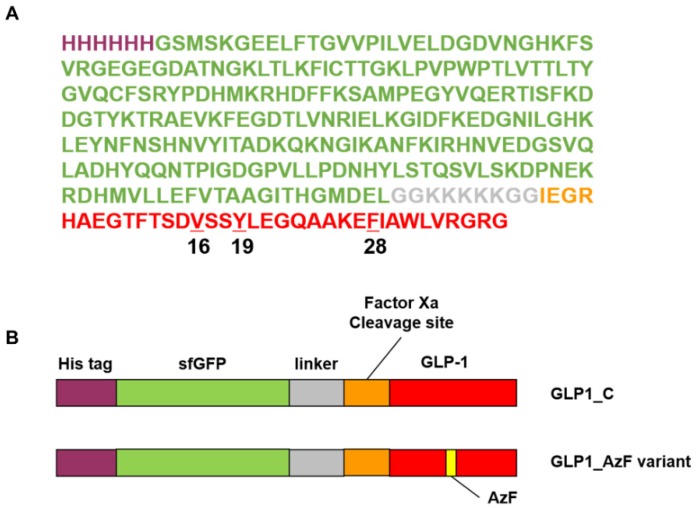
Construction of the sfGFP-fused GLP_C (sfGFP-GLP1_C) and sfGFP-fused GLP1_AzF (sfGFP-GLP1_AzF) variants. (**A**) The amino acid sequence of sfGFP-GLP1_C. The polyhistidine tag, sfGFP, linker, Factor Xa cleavage site, and GLP1_C are indicated by purple, green, gray, orange, and red lettering, respectively. The p-azido-l-phenylalanine (AzF) insertion sites are numbered in black. (**B**) Annotated protein features of the sfGFP-GLP1_C and sfGFP-GLP1_AzF variants. The polyhistidine tag (His tag), sfGFP, linker, Factor Xa cleavage site, and GLP1_C are indicated by purple, green, gray, orange, and red, respectively. The AzF insertion position is indicated by yellow.

In the present study, we chose glucagon-like peptide-1 (GLP-1)—which is widely used for the treatment of type 2 diabetes—as a model therapeutic peptide. GLP-1 is a 31-mer peptide (residues 7 to 37) with a very short half-life (approximately 3 min) [43]. It is secreted by pancreatic L-cells and exerts an important function on the regulation of serum glucose levels through multiple mechanisms in the liver, muscles, pancreas, and the central nervous system [44]. GLP-1 is known to enhance insulin secretion through signaling pathways including cAMP upregulation, resulting from GLP-1R activation upon GLP-1 binding [45]. Specifically, we used a GLP-1 variant with a lysine-to-arginine mutation at position 34 (GLP1_C). To obtain recombinant GLP1_C from *E. coli*, we fused GLP1_C to sfGFP using a linker containing the IEGR sequence cleaved by Factor Xa (Figure 3). GLP-1 becomes therapeutically effective when it binds its receptor (GLP-1R). Given that a large portion of GLP-1 is involved in GLP-1R binding, we speculated that the site of albumin conjugation would affect the efficacy of GLP-1. To investigate the dependence of GLP-1 efficacy on the albumin conjugation site, we chose three sites on GLP-1—valine16 (V16), tyrosine19 (Y19), and phenylalanine28 (F28)—for the incorporation of AzF; those sites were therefore available for HSA conjugation. The three chosen residues (V16, Y19, and F28) have very hydrophobic sidechains. Therefore, we expected minimal perturbation of the folded structure of GLP-1 following the incorporation of hydrophobic AzF. As shown in Figure 3, we prepared three GLP-1 variants containing AzF (GLP1_16AzF, GLP1_19AzF, and GLP1_28AzF), and carried out site-specific conjugation of HSA to generate the HSA-GLP1 conjugates. We subsequently investigated the serum half-lives and efficacies of the three HSA-GLP1 conjugates.

## 2. Materials and Methods

### 2.1. Materials

We purchased p-azido-l-phenylalanine (AzF; purity > 99%) from Chem-Impex International (Wood Dale, IL, USA). Nickel–nitrilotriacetic acid (Ni-NTA) agarose and propylene columns were obtained from Qiagen (Valencia, CA, USA). We purchased dibenzocyclooctyne-PEG_4_-maleimide (DBCO-PEG_4_-MAL; purity > 95%) and dibenzocyclooctyne-PEG_4_-carboxyrhodamine 110 (DBCO-PEG_4_-carboxyrhodamine; purity > 95%) from Click Chemistry Tools LLC (Scottsdale, AZ, USA). Disposable PD-10 desalting columns, HiTrap Q HP anion exchange columns, and HiTrap SP HP cation exchange columns were purchased from GE Healthcare (Little Chalfont, Buckinghamshire, UK). We purchased Vivaspin centrifugal concentrators (each with a molecular weight cut-off of 10 kDa) from Sartorius (Weender Landstraße, Göttingen, Germany). The GLP1_C peptide was synthesized by GenScript (Piscataway, NJ, USA). Anti-GLP-1 monoclonal antibody was purchased from Thermo Fisher Scientific (Waltham, MA, USA). Fetal bovine serum and antibiotic-antimycotic for cell culture were purchased from Gibco (Gaithersburg, MD, USA). X-tremeGENE HP DNA transfection reagent was purchased from Roche Diagnostics GmbH (Mannheim, Germany). cAMP Parameter Assay Kit was purchased from R&D Systems (Minneapolis, MN, USA). Iscove’s modified Dulbeco’s medium and all other chemicals (at least ACS reagent grade) were purchased from Sigma-Aldrich unless otherwise indicated.

### 2.2. Expression and Purification of sfGFP and GLP-1 Fusion Protein

We sub-cloned the DNA fragment encoding GLP1_C with the Factor Xa cleavage site (IEGR) and the linker (GGKKKKKGG) into the C-terminus of sfGFP in the pQE80-sfGFP plasmid using a DNA synthesis kit from Macrogen, Inc. (Seoul, South Korea) and standard cloning techniques to generate plasmid pQE80-sfGFP-GLP1_C. For the site-specific incorporation of AzF, an amber codon was introduced into the V16, Y19, or F28 sites of GLP-1 by site-directed PCR mutagenesis using the pQE80-sfGFP-GLP1_C plasmid as a template and the primers described in Table S1. In each of the three GLP-1 variants, the alanine at position 8 was also mutated to glycine to enhance its resistance to dipeptidyl peptidase IV (DPP IV)-mediated proteolysis by site-directed PCR mutagenesis using primers A8G_F and A8G_R (Table S1), generating three pQE80-sfGFP-GLP1_Amb plasmids (pQE80-sfGFP-GLP1_16Amb, pQE80-sfGFP-GLP1_19Amb, and pQE80-sfGFP-GLP1_28Amb). The pEvol-pAzFRS.1.t1 plasmid—which encodes the orthogonal pair MjTyrRS/MjtRNACUA that is specific for AzF—was obtained from Addgene (Addgene plasmid # 73547) [41].

We co-transformed two plasmids (pEvol-pAzFRS.1.t1 and one of the three pQE80-sfGFP-GLP1_Amb plasmids) into C321ΔA.exp. *E. coli* cells (Addgene # 49018) [42]. The transformed cells were cultured overnight with ampicillin on a Luria broth (LB) agar plate at 37 °C. We then picked a single colony from the plate and pre-cultured it overnight in 3 mL of fresh LB medium with 100 μg/mL ampicillin and 35 μg/mL chloramphenicol at 37 °C while shaking at 210 rpm. The cultured cells were inoculated into 200 mL of fresh 2x YT medium with 100 μg/mL ampicillin and 35 μg/mL chloramphenicol at 37 °C while shaking at 210 rpm. When the optical density at 600 nm (OD600) reached 0.4, we added a solution of AzF in 0.2 M NaOH to the culture to produce a final concentration of 1 mM. After 30 min, we added 1 mM IPTG and 0.2% (*w*/*v*) l-(+)-arabinose for induction, and further incubated the cells overnight at 25 °C. We centrifuged the cultured cells at 6000 rpm for 15 min, and stored them as wet pellets at −80 °C.

We purified the sfGFP-GLP1 fusion protein variants that contained AzF (sfGFP-GLP1_AzF) by affinity chromatography using Ni-NTA resin with an affinity to the hexahistidine tag of sfGFP. Cell lysis was achieved by incubating the stored pellets with 0.5 mg/mL lysozyme in 20 mL of 50 mM phosphate buffer (pH 7.5) containing 300 mM NaCl and 10 mM imidazole, and then sonicating the mixture for 48 min (10 s on/20 s off cycle). We centrifuged the cell lysate at 12,000 g for 20 min, then incubated the supernatant with Ni-NTA resin on ice for 40 min. The mixture was then poured into a polypropylene column. We washed the column with 50 mM phosphate buffer (pH 7.5) containing 300 mM NaCl and 20 mM imidazole, then eluted the protein with 50 mM phosphate buffer (pH 7.5) containing 300 mM NaCl and 250 mM imidazole. Further purification was conducted by anion exchange chromatography using a HiTrap Q HP column in 20 mM Tris buffer (pH 8.0).

### 2.3. Preparation of HSA Conjugate of GLP-1

We dissolved lyophilized HSA obtained from human serum (Sigma-Aldrich, A3782), in 20 mM bis-Tris (pH 7.0), and purified it by anion exchange chromatography using a HiTrap Q HP column with 20 mM bis-Tris (pH 7.0) to remove high-molecular-weight impurities. The purified HSA was then reacted with DBCO-PEG4-MAL in a molar ratio of 1:4 for 3 h, then desalted with 20 mM Tris (pH 8.0). We reacted 20 μM sfGFP-GLP-1_AzF and HSA-DBCO in a molar ratio of 1:2 overnight at room temperature (RT), and buffer-exchanged the mixture into 20 mM sodium phosphate buffer (pH 6.0). The HSA-sfGFP-GLP-1 conjugate was separated from the unreacted HSA-DBCO and sfGFP-GLP-1 by cation exchange chromatography using a HiTrap SP HP column.

The separated conjugate was buffer-exchanged into Factor Xa reaction buffer (20 mM Tris; 2 mM CaCl_2_; 10 mM NaCl; pH 8), and concentrated to 10 μM using a Vivaspin centrifugal concentrator. To cleave the sfGFP region, we incubated the conjugated protein with 1/500 (*w*/*w*) Factor Xa protease at RT for 18 h, then added 0.1 mg/mL dansyl-Glu-Gly-Arg-chloromethyl ketone and Factor Xa inhibitor in a molar ratio of 0.7. The Factor Xa-processed conjugate solution was buffer-exchanged into 20 mM bis-Tris buffer (pH 6.0), and the GLP1_HSA variant was purified by anion exchange chromatography using a HiTrap Q HP column. Except for sfGFP-GLP-1, we quantified all the proteins by measuring their absorbance at 280 nm using a Synergy™ microplate reader (BioTek, Winooski, VT, USA). We calculated the molar extinction coefficient at 280 nm using the following equation: ε280 = (5500 × εTrp) + (1490 × εTyr) + (125 × ε(disulfide bond)) + (2620 × εAzF) [46]. sfGFP-GLP-1 was quantified using a Pierce™ BCA Protein Assay Kit (Thermo Fisher Scientific, Inc., Waltham, MA, USA).

### 2.4. Mass Spectrometric Analysis

To determine their monoisotopic masses, we desalted GLP1_16AzF, GLP1_19AzF, and GLP1_28AzF using a ZipTip C18 system according to the manufacturer’s protocol. The desalted peptides were mixed with a 1:1 (*v*:*v*) mixture of α-cyano-4-hydroxy cinnamic acid (HCCA) saturated TA30 (a solution comprising 30% acetonitrile and 0.1% trifluoroacetic acid), and applied to a polished steel plate, then subjected to mass characterization by microflex matrix-assisted laser desorption/ionization time-of-flight mass spectroscopy (MALDI-TOF; Bruker Daltonics, Bremen, Germany).

To determine their intact masses, we desalted GLP1_16HSA, GLP1_19HSA, and GLP1_28HSA on a ZipTip C18 system according to the manufacturer’s protocol. The desalted conjugates were mixed with a 1:1 (*v*:*v*) mixture of DHB matrix (20 mg/mL 2,5-dihydroxybenzoic acid in TA30), and applied to a polished steel plate, then subjected to mass characterization by microflex MALDI-TOF (Bruker Daltonics, Bremen, Germany). The mass spectrum of each sample was obtained using flexControl autoflex TOF/TOF software (Bruker Daltonics, Bremen Germany).

### 2.5. Labeling of sfGFP-GLP1_AzF by SPAAC

We reacted the sfGFP-GLP1_16AzF variants (20 μM) with DBCO-PEG4-carboxyrhodamine (60 μM) in phosphate-buffered saline at RT for 2 h, then subjected the products to sodium dodecyl sulfate–polyacrylamide gel electrophoresis (SDS-PAGE) to measure the in-gel fluorescence using a Bio-Rad ChemiDocTM XRS+ system (Hercules, CA, USA). The emitted light (>510 nm) was captured following illumination at λex = 302 nm.

### 2.6. In Vitro Activity ASSAY

We determined the in vitro activity of the GLP1_C and GLP1_HSA variants by measuring the cAMP production associated with human GLP-1R using HEK-293 cells overexpressing GLP-1R. 5 × 10^5^ HEK-293 cells (ATCC, CRL1573, Manassas, VA, USA) per well were seeded on 48-well plates using Iscove’s modified Dulbeco’s medium containing 10% fetal bovine serum and 1% antibiotic-antimycotic. After 16 h incubation, cells were transfected with the plasmid pcDNA3.1-GLP-1R_tango (Addgene, Cambridge, MA, USA) [47] using DNA transfection reagent in serum free media at 37 °C and 5% CO_2_. After 48 h transfection, The GLP1_C and GLP1_HSA variants at concentrations 10 times higher than the final concentration were diluted with medium containing 10% serum and treated in each well for 15 min before the cells were lysed. We determined the amount of cAMP in the cell lysates using a cAMP Parameter Assay Kit (R&D Systems, Minneapolis, MN, USA), and converted the result into % activity based on the amount of cAMP detected in cells treated with 10^−7^ M GLP1_C. For each of the GLP1_C and GLP1_HSA variants, the graph of % activity against the logarithmic concentration of the variant was fitted to the dose–response curve, and the half maximal effective concentration (EC50) was calculated using OriginPro (OriginLab, Northampton, Massachusetts, USA) software. The synthetic peptide GLP1_C was dissolved in H_2_O and stored at −20 °C. Before carrying out the assay, we slowly thawed out the peptide at 4 °C and diluted it with phosphate-buffered saline to obtain the required concentration.

### 2.7. In Vitro GLP-1-Albumin Enzyme-Linked Immune Sorbent Assay

We coated the immunoplate with polyclonal anti-albumin rabbit antibody in coating buffer (0.1 M bicarbonate buffer; pH 9.6) at 4 °C overnight, and blocked the coated plate with 5% skim milk at 37 °C for 1 h. Predetermined concentrations of each GLP1_HSA variant standard and collected serum samples were diluted in the same buffer and incubated in each well at RT for 2 h. The diluted monoclonal anti-GLP-1 mouse antibody in the blocking solution was incubated at RT for 2 h. An anti-mouse IgG antibody-horseradish peroxidase (HRP) conjugate was diluted in the blocking solution, and the mixture was incubated at RT for 1 h. We then reacted 100 μL of the substrate for 5 min in each well. The reaction was stopped by adding 2 M H_2_SO_4_, and the absorbance of each well was measured at 450 nm using a Synergy™ microplate reader (BioTek, Winooski, VT, USA).

### 2.8. Pharmacokinetic Studies of GLP-1-HSA Conjugates

We carried out in vivo pharmacokinetic studies on eight-week-old female BALB/c mice, which were purchased from DBL. The mice had free access to water and food and were maintained in a 12-h light/12-h dark cycle. All animal procedures were performed in accordance with the Guidelines for Care and Use of Laboratory Animals proposed by the Gwangju Institute of Science and Technology (GIST), and were approved by the Animal Ethics Committee of GIST (Approval numbers: GIST-2019-022 (21 May 2019) and GIST-2019-071 (4 October 2019)).

The mice were randomly divided into three groups (*n* = 5) and were weighed before injection. GLP1_HSA variants (10 nmol/kg dose) were intravenously administrated to each group of mice. Blood samples (below 70 μL) were collected from the retroorbital venous sinus at 0.16, 1, 2, 4, 8, 12, and 24 h after administration. We obtained the serum by centrifuging the blood samples at 2500 rpm for 10 min at 4 °C, and stored it at −20 °C until it was required. The serum concentration of the GLP1_HSA conjugate at each time-point was measured by carrying out three enzyme-linked immunosorbent assays (ELISAs).

### 2.9. In Vivo Intraperitoneal Glucose Tolerance Test (IPGTT)

We performed in vivo intraperitoneal glucose tolerance tests (IPGTTs) on seven-week-old male C57BL/6J mice, which we purchased from DBL. The mice were randomly divided into five groups (*n* = 6). The IPGTTs were performed after glucose (1.5 g/kg dose) had been intraperitoneally administered to C57BL/6J male mice that had been fasted for 3 h. We obtained blood samples from the tail of each mouse at the predetermined time using an Accu-Check Guide (Roche Diabetes Care, Indianapolis, IN, USA). GLP1_C (100 nmol/kg dose), each of the GLP1_HSA variants (100 nmol/kg dose), or saline was subcutaneously administrated 1 h before glucose injection.

## 3. Results and Discussion

### 3.1. Site-Specific Incorporation of AzF into V16, Y19, or F28 Site of sfGFP-GLP1 Fusion Protein

In the GLP-1 variant used in the present study (GLP1_C), the arginine at position 34 replaced the lysine in the wild-type GLP-1. This K34R mutant has comparable biological activity to that of wild-type GLP-1 [48]. Furthermore, to ensure resistance to the ubiquitous protease dipeptidyl peptidase IV (DPP-IV), alanine at position 8 was mutated to glycine in GLP1_C, in addition to AzF incorporation (Figure 3) [49].

We chose sites for AzF incorporation based on the solvent accessibility and hydrophobicity of the sites, as previously reported [37,50]. Phenylalanine, tryptophan, and tyrosine were chosen as primary sites for AzF incorporation. However, we avoided those residues at the N-terminus of GLP1_C, which, according to an evaluation of the GLP-1 structure complexed with GLP1-R (PDB ID: 5vai), is buried inside GLP1-R. Therefore, we selected two sites: tyrosine at position 19 and phenylalanine at position 28. We also selected valine at position 16, because the mutation of valine to tyrosine at position 16 does not significantly reduce the activity of GLP-1 according to the structure-activity relationship studies [51,52,53]. The reassignment of the UAG codon to AzF was successfully achieved by genetically engineered *E. coli* C321ΔA.exp expressing the engineered pair MjTyrRS/MjtRNACUA [41,42], generating three sfGFP-GLP1_AzF variants: sfGFP-GLP1_16AzF, sfGFP-GLP1_19AzF, and sfGFP-GLP1_28AzF. sfGFP-GLP1_C without any AzF was expressed in TOP10 *E. coli* cells.

**Figure 4:**
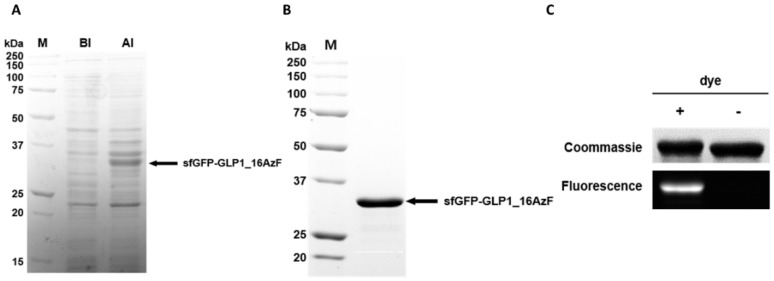
Site-specific incorporation of AzF into recombinant GLP-1 fused to sfGFP. (**A**) Coomassie Brilliant Blue-stained sodium dodecyl sulfate–polyacrylamide gel electrophoresis (SDS-PAGE) image of cell lysates before (BI) and after (AI) induction; the prominent band attributable to the sfGFP-GLP-1 fusion protein is indicated (arrow). Protein molecular weight standards are shown in lane M. (**B**) Purified sfGFP-GLP1_16AzF on Coomassie Brilliant Blue-stained SDS-PAGE gel after nickel–nitrilotriacetic acid (Ni-NTA) chromatography and anion exchange chromatography (arrow). Protein molecular weight standards are shown in lane M. (**C**) Protein gel image of sfGFP-GLP1_16AzF treated with or without fluorescent dye (DBCO-PEG4-carboxyrhodamine). The gel was subjected to UV irradiation (302 nm) to excite the fluorophore (fluorescence) and stained with Coomassie Brilliant Blue (Coomassie) to visualize the protein.

When we compared cell lysates obtained before (BI) and after (AI) induction with sfGFP-GLP1_16AzF, we noticed a new band attributable to the AI sample in the Coomassie Brilliant Blue-stained protein gel (Figure 4A). The band was located between 25 and 37 kDa, which matched the expected molecular weight of the sfGFP-GLP1_16AzF fusion protein (approximately 32 kDa) (Figure 4A). We obtained the purified sfGFP-GLP1_16AzF by Ni-NTA chromatography and anion exchange chromatography (Figure S1 in Supplementary Materials). A band between 25 and 37 kDa attributable to purified sfGFP-GLP1_16AzF was also present in the Coomassie Brilliant Blue-stained protein gel (Figure 4B). Since the expression and purification of each sfGFP-GLP1_AzF variant was carried out in the same way, the expression and purification of sfGFP-GLP1_16AzF was described in detail as a representative case. The production yield of purified sfGFP-GLP1_16AzF was 33.6 mg/L in the basis of culture volume, demonstrating the convenient production of GLP-1 in a good yield even without process optimization. The optimized cell culture led to over 500 mg/L production yield of human growth hormone containing a non-natural amino acid at a 1000 L scale [54]. Considering that the cleavage efficiency of the fusion protein using Factor Xa is usually high, these results show that the production of a therapeutic recombinant peptide containing a non-natural amino acid is a feasible option for practical peptide preparation.

We carried out fluorescent dye labeling through the SPAAC reaction to confirm the incorporation of AzF in sfGFP-GLP1_16AzF and determine its reactivity. DBCO-carboxyrhodamine 110 was reacted with sfGFP-GLP1_16AzF, and the in-gel fluorescence of the reaction mixture was determined. Bands attributable to sfGFP-GLP1_16AzF were clearly visible in the Coomassie Brilliant Blue-stained protein gel (Figure 4C). The sfGFP-GLP1_16AzF that reacted with the fluorescent dye was highly fluorescent, indicating successful SPAAC reaction between sfGFP-GLP1_16AzF and DBCO-carboxyrhodamine 110 (Figure 4C).

The incorporation of AzF was also verified by MALDI-TOF mass spectroscopy (MS). As the expected monoisotopic mass of sfGFP-GLP1_C is large (i.e., 32544.3 Da), we anticipated that the evaluation of the monoisotopic mass of sfGFP-GLP1_AzF by MALDI TOF would be inaccurate. Therefore, we cleaved sfGFP from the sfGFP_GLP1 fusion protein using the proteolytic enzyme Factor Xa to determine the masses of GLP1_C and the GLP1_AzF variants to confirm AzF incorporation. The masses of the GLP1 variants were determined by MALDI-TOF MS analysis. The monoisotopic mass of GLP1_C was 3382.7 m/z, which closely matched the theoretical mass of GLP1_C (3382.7 m/z) (Figure S2). The monoisotopic masses of GLP1_16AzF, GLP1_19AzF, and GLP1_28AzF were 3430.5, 3367.5, and 3383.3 m/z, respectively (Figure S2), which closely matched the expected masses (3431.6, 3367.6, and 3383.6 m/z, respectively) with less than 0.15% error. Furthermore, the mass differences between GLP1_C and the GLP1_AzF variants (GLP1_16AzF, GLP1_19AzF, and GLP1_28AzF) were 47.8, −15.2, and −0.6 Da, respectively, which closely matched the expected values (48.5, −15.1, and −0.9 Da, respectively). We additionally confirmed the molecular weight of GLP1_C on the protein gel (Figure S3). These results indicated the successful site-specific incorporation of clickable AzF into GLP-1 fused with sfGFP in *E. coli*, and that the AzF incorporated in the GLP-1 retained its reactivity.

### 3.2. Preparation of Site-Specifically Albuminconjugated GLP1_HSA Variants

**Figure 5:**
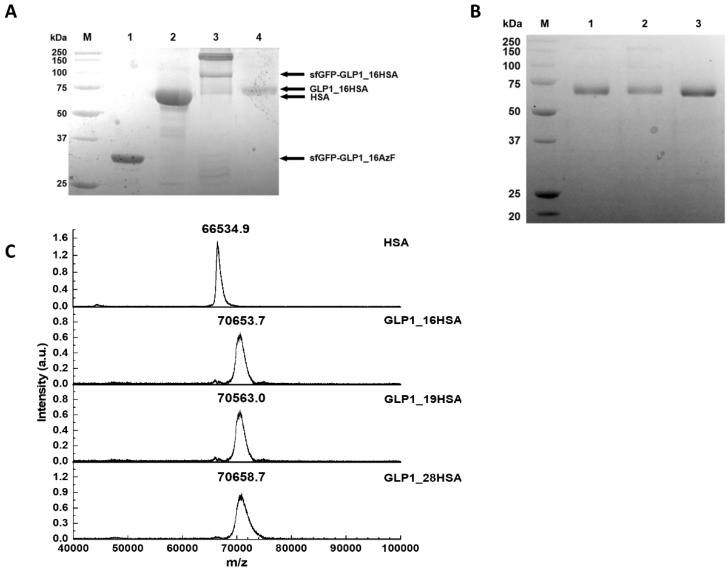
Confirmation of GLP1_HSA variants and their intermediates during production. (**A**) Image of the Coomassie Brilliant Blue-stained gel indicating the presence of the protein intermediates of GLP1_HSA production. GLP1_16HSA is presented as a representative of the GLP1_HSA variants. Protein molecular weight standards (lane M), sfGFP-GLP1_16AzF (lane 1), purified HSA (lane 2), sfGFP-GLP1_16HSA (lane 3), and GLP1_16HSA (lane 4). In lane 3, the bands between 150 and 250 kDa are attributable to aggregates. (**B**) Coomassie Brilliant Blue-stained protein gel of the purified GLP1_16HSA (lane 1), GLP1_19HSA (lane 2), and GLP1_28HSA (lane 3). Protein molecular weight standards are shown in lane M. (**C**) Matrix-assisted laser desorption/ionization time-of-flight mass spectroscopy (MALDI-TOF MS) analysis of GLP1_HSA variants to determine their intact masses (a.u. indicates arbitrary unit).

We used a heterobifunctional linker, DBCO-PEG_4_-MAL, to append DBCO functionality to HSA, generating HSA-PEG_4_-DBCO. HSA has one free cysteine residue at position 34 (C34), which has been utilized for protein conjugation via a thiol–maleimide reaction, because it is located away from the FcRn-binding domain [37]. The DBCO in HSA-PEG_4_-DBCO reacted with the azide functional group of AzF in the GLP1_16AzF variant via SPAAC, generating the sfGFP-GLP1_16HSA variant (Figure 5A). After isolation of sfGFP-GLP1_16HSA by cation exchange chromatography (Figure S4), the conjugate was processed with Factor Xa to produce the GLP1_16HSA variant. Finally, GLP1_16HSA was purified using anion exchange chromatography (Figure 5A and Figure S5). The same procedures were used to obtain GLP1_19HSA and GLP1_28HSA. There were conspicuous bands between 50 and 75 kDa (approximately 70 kDa) in the Coomassie Brilliant Blue-stained protein gel that were attributable to the purified GLP1_HSA variants (GLP1_16HSA, GLP1_19HSA, and GLP1_28HSA) (Figure 5B).

The purified GLP1_HSA variants were subjected to MALDI-TOF MS analysis. The masses of HSA, GLP1_16HSA, GLP1_19HSA, and GLP1_28HSA were 66534.9, 70653.7, 70563.0, and 70658.7 m/z, respectively (Figure 5C). The values were very close to the expected values (66473.2, 70606.8, 70542.8, and 70558.8 m/z) with a very minor error rate (less than 0.15%). The protein gel and MALDI-TOF MS analysis results demonstrated that three GLP_HSA variants were successfully prepared.

### 3.3. In Vivo Study

**Figure 6:**
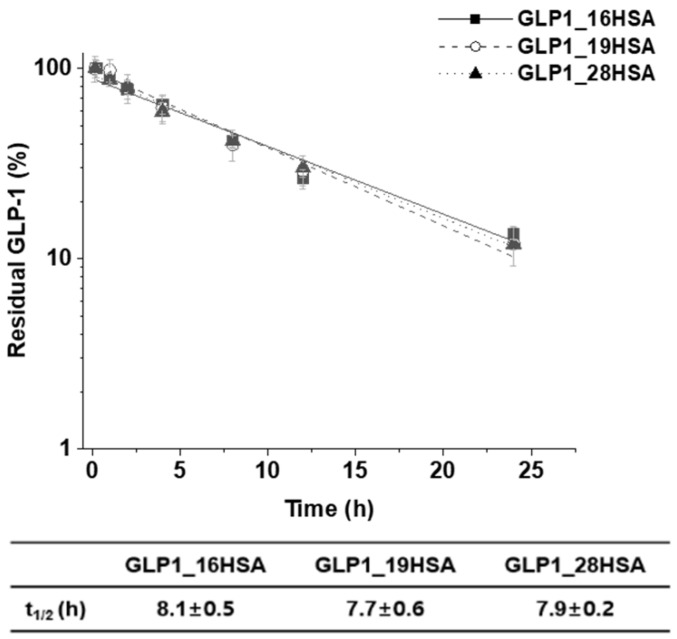
Pharmacokinetic profiles of the GLP1_HSA variants in mice. Each data point in the graph represents the mean ± SD (*n* = 5). The logarithmic serum concentrations over time were plotted to produce a linear fit.

We investigated the pharmacokinetic profiles of GLP1_16HSA, GLP1_19HSA, and GLP1_28HSA to determine their extended half-lives. Each GLP1_HSA variant was intravenously administered to the BALB/c mice. After administration, blood samples were collected at seven time-points within 24 h. The serum concentration at each time-point was measured by a sandwich ELISA targeting both HSA and GLP-1 (Figure 6). The half-lives of GLP1_16HSA, GLP1_19HSA, and GLP1_28HSA were 8.4, 7.4, and 8.0 h, respectively. The half-life of the GLP1_HSA variants increased by approximately 160 times compared to the wild-type GLP-1 (3 min), and all three variants had similar values [43]. These results indicate that HSA conjugation successfully extended the serum half-lives of the GLP-1 variants.

The extended half-lives of the variants correspond with those reported in a previous study. The extended half-lives obtained in our study are in accordance with one (8.5 h) reported for chemically synthesized GLP-1 conjugated to HSA in mice [32]. PEG-conjugation to GLP-1 resulted in 1.7 or 12.1 h of serum half-life in rat depending on the molecular weight of PEG molecules used. Considering that the serum half-life of one drug in rats is longer than that in mice, the serum half-life extension by HSA conjugation was more effective than PEG conjugation [55]. Furthermore, it is noteworthy that the serum half-life extension of HSA-conjugated GLP-1 in humans would be greater than that in mice, because HSA is known to have a much stronger binding affinity toward human FcRn than to mouse FcRn [38].

**Figure 7:**
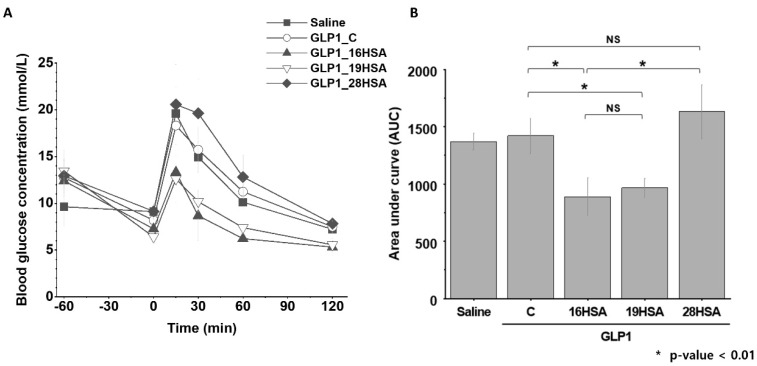
Glucose profiles of saline, GLP1_C, and GLP1_HSA variants determined by intraperitoneal glucose tolerance tests (IPGTTs). The IPGTT glucose profiles of saline (the negative control), GLP1_C (the positive control), and the GLP1_HSA variants were determined to evaluate the in vivo activity. (**A**) GLP1_C and each of the GLP1_HSA variants (100 μL volume; 100 nmol/kg dose) were subcutaneously injected into the mice in each group (*n* = 6) 60 min before the intraperitoneal injection of glucose (1.5 g/kg dose; 0 min). The blood glucose concentration at protein injection (−60 min), at glucose injection (0 min), and at 15, 30, 60, and 120 min after glucose injection were recorded. Each data point on the graph represents the mean ± SD (*n* = 6). (**B**) The area under the curve (AUC) of each graph from 0 to 120 min was calculated and compared in the graph. The mean AUCs ± SDs are presented (*n* = 6). NS: not significant; * *p*-value < 0.01.

To evaluate the in vivo activities of GLP-1_HSA variants, an intraperitoneal glucose tolerance test (IPGTT) for each variant was performed on C57BL/6J mice (Figure 7). As for the negative control group (saline), the blood glucose sharply increased upon the injection of glucose but returned to the normal level at 120 min after the injection (Figure 7). The blood glucose profiles of GLP1_C and GLP1_28HSA groups were very similar to that of the negative control group, indicating that neither GLP1_C nor GLP1_28HSA showed any substantial glucose-lowering effect (Figure 7A). The trend in AUC values of the negative control, GLP1_C, and GLP1_28HSA were consistent with that in glucose profiles (Figure 7B). Such a poor glucose-lowering effect of GLP1_C can be attributed to its short half-life. In contrast to GLP1_28HSA, the blood glucose levels of both GLP1_16HSA and GLP1_19HSA groups were substantially lower than those of the negative control and GLP1_C (Figure 7A). Similarly, the AUC values of both GLP1_16HSA and GLP1_19HSA were significantly lower than those of the negative control and GLP1_C (Figure 7B). These results implied that both GLP1_16HSA and GLP1_19HSA variants have greater glucose-lowering activity compared to GLP1_28HSA and exhibit a more prolonged glucose-lowering activity than that of GLP1_C. Considering that the serum half-life of GLP1_28HSA was much longer than that of GLP1_C, similar glucose profiles of GLP1_C and GLP_28HSA indicated that the glucose-lowering activity of GLP1_28HSA was extremely low compared to that of GLP1_C. To explain the different glucose-lowering effects of GLP1_HSA variants, GLP1_C and GLP1_HSA variants were subjected to in vitro activity assays.

### 3.4. In Vitro Activity Assay

**Figure 8:**
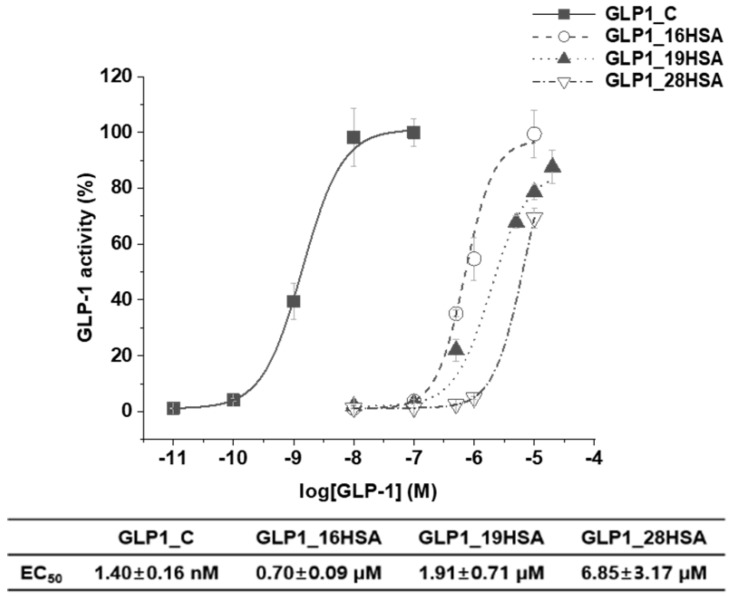
In vitro biological activities of GLP1_C, GLP1_16HSA, GLP1_19HSA, and GLP1_28HSA determined in GLP-1R-expressing cells. GLP-1R-expressing HEK cells were treated with increasing concentrations of GLP1_C, GLP1_16HSA, GLP1_19HSA, or GLP1_28HSA. The *y*-axis values were converted to percentages by averaging the cAMP production of 10^−7^ M GLP1_C. Each data point represents the mean (*n* = 3) ± SD.

To evaluate the biological activities of the recombinant GLP1_C and GLP1_HSA variants, cAMP production of HEK-293 cells overexpressing GLP-1R was measured in vitro. First, the biological activity of recombinant GLP1_C was not significantly different from that of chemically synthesized GLP1_C (Figure S6). Second, EC50 values of GLP1_C, GLP1_16HSA, GLP1_19HSA, and GLP1_28HSA were 1.4 nM, 0.70 μM, 1.91 μM, and 6.85 μM, respectively (Figure 8). Therefore, the biological activities of GLP1_HSA variants were in decreasing order of GLP1_16HSA, GLP1_19HSA, and GLP1_28HSA, which was consistent with the trend in blood glucose profiles.

GLP-1R—a class B G-protein-coupled receptor—is a transmembrane protein comprising two domains: an N-terminal extracellular domain (ECD) and a transmembrane domain (TMD) [56]. A previous study has reported that both the C- and N-termini of GLP-1 should interact with the ECD and TMD of GLP-1R, respectively, to activate GLP-1R in the cell membrane [57]. Other studies have published that the F28 site of GLP-1 is important for the retention of its biological effects, because it interacts with the ECD of GLP-1 [58,59]. In the present study, the GLP1_28HSA variant had a markedly high EC_50_ value, i.e., 10-fold higher than that of GLP1_16HSA. The low GLP1_28HSA activity can be attributed to compromised interactions between GLP-1 and the ECD of GLP-1R, because the site was used for conjugation to HSA. Furthermore, the HSA conjugated to the F28 of GLP-1 could have sterically hindered the binding of GLP1-R and GLP1_28HSA. However, previous reports suggest that neither V16 nor Y19 on GLP-1 interact significantly with the ECD or TMD of GLP-1R [58,59,60]. Therefore, the steric hindrance between GLP-1R and GLP1_16HSA or GLP1_19HSA could be weaker than the steric hindrance between GLP-1R and GLP1_28HSA. This explains why the EC_50_ values of GLP1_16HSA and GLP1_19HSA were lower than that of GLP1_28HSA.

In a study by Bukrinski et al., a GLP-1-HSA conjugate—in which HSA was chemically conjugated to the C-terminus of GLP-1—produced a 3300-fold increase in the EC_50_ compared to the wild-type GLP-1 [32]. However, in the present study, GLP1_16HSA produced a 500-fold increase in the EC_50_ value, indicating the higher biological activity than that of the C-terminus HSA-conjugated GLP-1. We speculated that the difference between the EC_50_ values of GLP1_16HSA and GLP1_19HSA may result from the different position and direction of the HSA-conjugated to GLP-1, affecting the interaction with GLP-1R. The sidechain of the amino acid at position 16 is directed slightly upward, whereas the sidechain of the amino acid at position 19 is directed downward (Figure S7).

These results clearly demonstrate that specific site selection for HSA conjugation plays an important role in biological activity in vitro and blood glucose-lowering effects in vivo.

## 4. Conclusions

Using a suitable fusion tag, we successfully achieved the high expression of a small therapeutic peptide (GLP-1) in *E. coli*, which was not expressed well without the fusion tag. The production yield of the purified sfGFP-GLP1_AzF variant was over 30 mg/L. Considering almost complete removal of sfGFP from the fusion protein using Factor Xa, the recombinant production platform demonstrates the potential of convenient production of therapeutic peptides. We also successfully produced GLP-1 conjugated with HSA at specific sites by the site-specific incorporation of AzF using a bioorthogonal SPAAC reaction. Three HSA-conjugated GLP-1 variants were prepared to investigate the importance of the HSA conjugation site on GLP-1. The half-lives of all three GLP1_HSA conjugates (GLP1_16HSA, GLP1_19HSA, and GLP1_28HSA) increased substantially. Two of the three conjugates (GLP1_16HSA and GLP1_19HSA) exhibited predominant in vivo glucose-lowering activity compared to the unmodified GLP1_C. However, the biological activities of the HSA-conjugated peptide variants depended on the HSA conjugation site, implying that the HSA conjugation site of the therapeutic peptide affects its biological activity. Further optimization of the HSA conjugation site may generate GLP1_HSA variants with greater biological activity in vitro and blood glucose-lowering effect *in vivo*. Our results demonstrate a practical recombinant peptide platform for the extension of the serum half-life of therapeutic peptides produced by utilizing the site-specific conjugation of HSA. Despite a great potential of the strategy described here, it may have limitations. HSA-conjugation extends the serum half-life of therapeutic peptides but may reduce their efficacy, partially due to the restricted conformational change of therapeutic peptides upon HSA conjugation. Therefore, HSA-conjugation may not be very effective for quite flexible therapeutic peptides. Furthermore, HSA-conjugated therapeutic peptides may not across biological barriers such as blood vessel wall or blood-brain barrier as much as unmodified ones. Therefore, HSA-conjugation strategy may not be suitable for therapeutic peptides that should across biological barriers for their therapeutic efficacy.

## Supplementary Materials

The following are available online at https://www.mdpi.com/1999-4923/12/4/364/s1, Table S1: Oligonucleotide primers used in this study, Figure S1: Purification of sfGFP_16AzF, Figure S2: Monoisotopic mass confirmation by matrix-assisted laser desorption/ionization time-of-flight mass spectroscopy (MALDI-TOF MS) analysis of the chemically synthesized GLP1_C, recombinant GLP1_C, and GLP1_AzF variants, Figure S3. Confirmation of the molecular weight of GLP1_C on the 15% tricine gel stained with Coomassie brilliant blue, Figure S4: Purification of sfGFP-GLP1_16HSA after the conjugation of sfGFP-GLP1_16AzF and HSA-DBCO by strain-promoted azide–alkyne cycloaddition (SPAAC), Figure S5: Purification of GLP1_16HSA after proteolytic cleavage by Factor Xa, Figure S6: Comparative study of the biological activity of chemically synthesized GLP1_C (GLP1_C) and recombinant GLP1_C in GLP-1R expressing cells, Figure S7: Location of V16, Y19 and F28 residues on Cryo-EM structure of the activated Glucagon-like peptide-1 receptor.
