# Supplementary material for: Recombinant Peptide Production Platform Coupled with Site-Specific Albumin Conjugation Enables a Convenient Production of Long-Acting Therapeutic Peptide
Source: Pharmaceutics. 2020 Apr 16;12(4):364. doi: 10.3390/pharmaceutics12040364 (PMC7238188; doi:10.3390/pharmaceutics12040364)
Supplement: Supplementary file 1 [file pharmaceutics-12-00364-s001.pdf]

pharmaceutics

Supplementary Materials: Recombinant Peptide Production Platform Coupled with Site-Specific Albumin Conjugation Enables a Convenient Production of Long-Acting Therapeutic Peptide

Mijeong Bak, Junyong Park, Kiyoon Min, Jinhwan Cho, Jihyoun Seong, Young S. Hahn, Giyoong Tae and Inchan Kwon

Table S1. Oligonucleotide primers used in this study.

Primer Name	Oligonucleotide Sequence (5′→3′)	Generated Plasmid
V16AzF_F	AAGGCACCTTTACCAGCGATTAGAGTAGCTATCTGGAAGG	pQE80-sfGFP-GLP1_16Amb
V16AzF_R	CCTTCCAGATAGCTACTCTAATCGCTGGTAAAGGTGCCTT	
Y19AzF_F	GCGATGTGAGTAGCTAGCTGGAAGGTCAGGC	pQE80-sfGFP-GLP1_19Amb
Y19AzF_R	GCCTGACCTTCCAGCTAGCTACTCACATCGC	
F28AzF_F	GTCAGGCGGCCAAAGAATAGATTGCCTGGCTGGTGC	pQE80-sfGFP-GLP1_28Amb
F28AzF_R	GCACCAGCCAGGCAATCTATTCTTTGGCCGCCTGAC	
A8G_F	GCATCGAAGGTAGGCATGGTGAAGGCACCTTTACCAG	-
A8G_R	CTGGTAAAGGTGCCTTCACCATGCCTACCTTCGATGC	

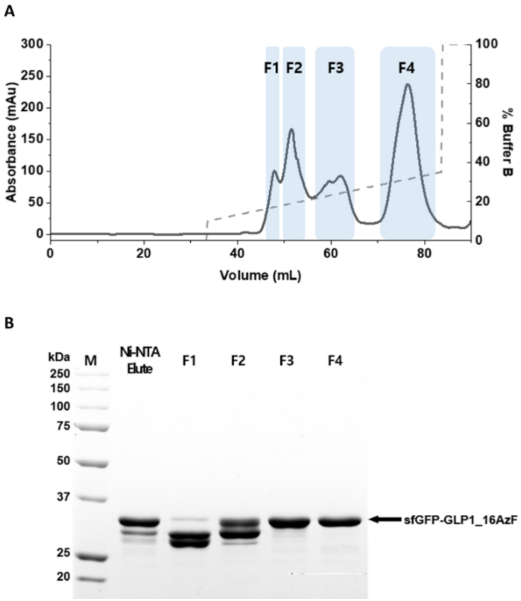

Figure S1. Purification of sfGFP-GLP1_16AzF. After nickel–nitrilotriacetic acid (Ni-NTA) purification of the cell lysates (the Ni-NTA elute in the gel image), further purification by anion exchange chromatography (**A**) was carried out to remove impurities from the sfGFP-GLP1_16AzF variant, as revealed by the gel image. Each fraction was investigated by sodium dodecyl sulfate–polyacrylamide gel electrophoresis (SDS-PAGE). (**B**) The elution sample from the Ni-NTA purification was desalted with PD-10 and loaded onto a HiTrap Q HP column equilibrated with 20 mM Tris (pH 8.0), then eluted with an NaCl gradient. The dotted line represents the percentage of Buffer B (20 mM Tris with 1 M NaCl; pH 8.0). sfGFP-GLP1_16AzF (fractions 3 and 4; F3 and F4) was isolated from the impurities expected from the premature transition termination caused by the "amber" stop codon (fractions 1 and 2; F1 and F2), as revealed by the protein gel.

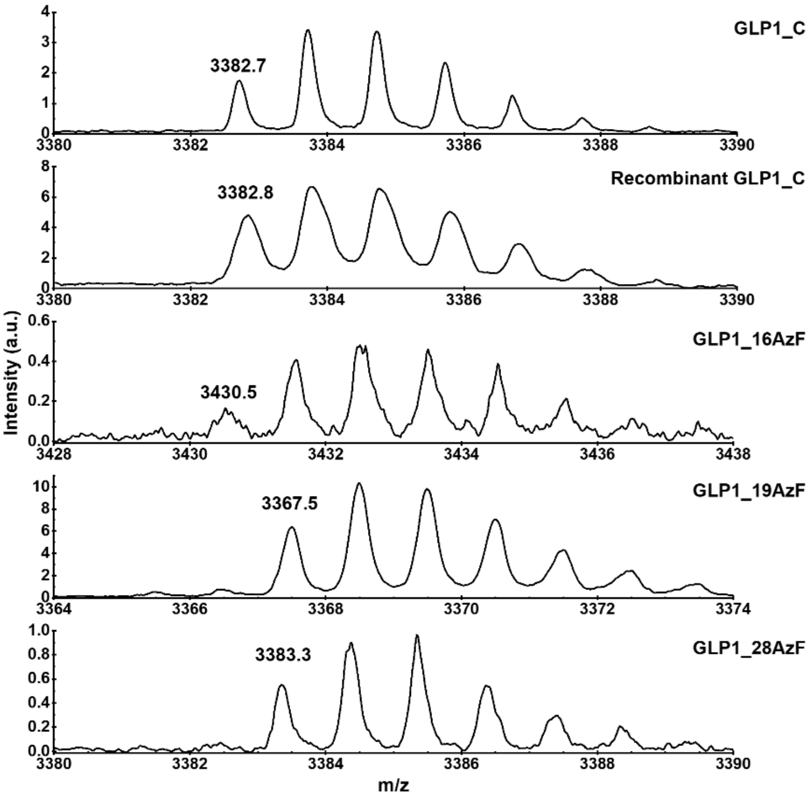

Figure S2. Monoisotopic mass confirmation by matrix-assisted laser desorption/ionization time-of-flight mass spectroscopy (MALDI-TOF MS) analysis of the chemically synthesized GLP1_C, recombinant GLP1_C, and GLP1_AzF variants. The monoisotopic masses of the chemically synthesized GLP1_C and recombinant GLP1_C were 3382.7 and 3382.8 m/z, respectively. The monoisotopic masses of GLP1_16AzF, GLP1_19AzF, and GLP1_28AzF were 3430.5, 3367.5, and 3383.3 m/z, respectively.

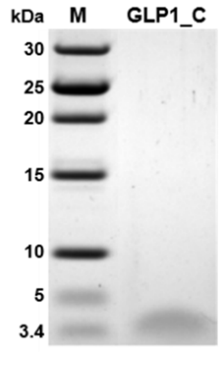

Figure S3. Confirmation of the molecular weight of GLP1_C on the 15% tricine gel stained with Coomassie brilliant blue. The band for GLP1_C located around 3.4-kDa band, which is consistent with its expected molecular weight, 3381.7 Da. M: protein molecular weight standards (Thermo Fisher Scientific, Inc., Waltham, MA, USA).

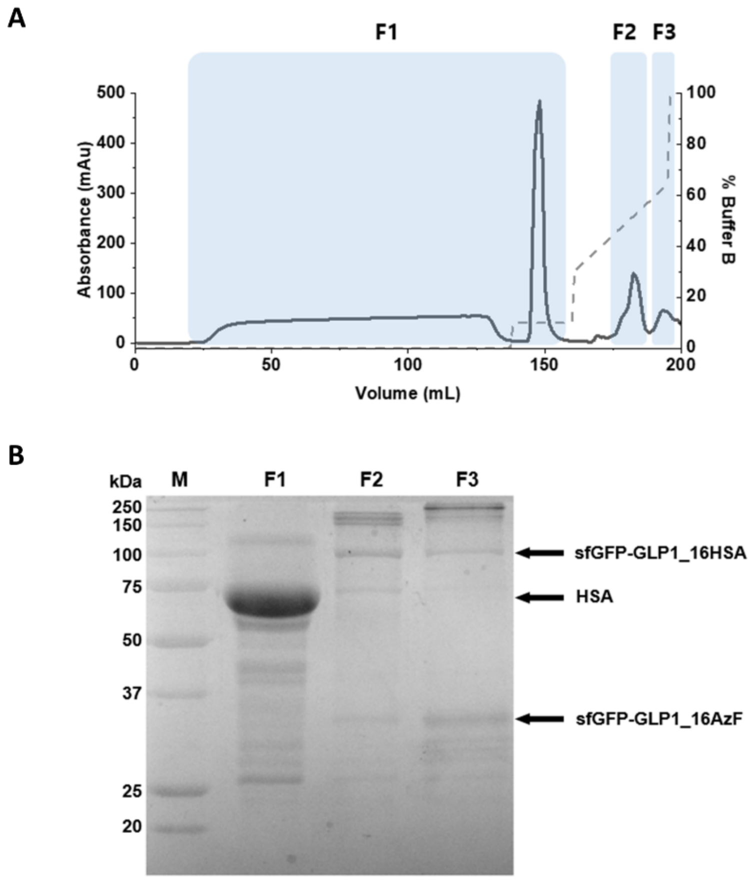

Figure S4. Purification of sfGFP-GLP1_16HSA after the conjugation of sfGFP-GLP1_16AzF and HSA-DBCO by strain-promoted azide–alkyne cycloaddition (SPAAC). After the conjugation of sfGFP-GLP1_16AzF and HSA-DBCO by SPAAC, cation exchange chromatography was carried out to purify the sfGFP-GLP1_16HSA. (**A**) The cation exchange chromatogram. The reacted conjugate was desalted with PD-10, loaded onto a HiTrap SP-HP column equilibrated with 20 mM sodium phosphate (pH 6.0), and eluted with an NaCl gradient. The fractions were collected and evaluated on protein gel (B). The dotted line represents the percentage of Buffer B (20 mM sodium phosphate with 1 M NaCl; pH 6.0). (**B**) Protein gel image associated with the cation exchange chromatogram. Protein molecular weight standards are shown in lane M. There was unreacted albumin-DBCO, which was not bound to the column (F1). The sfGFP-GLP1_16HSA conjugate was eluted in F2. The unreacted sfGFP-GLP1_16AzF was eluted in F3.

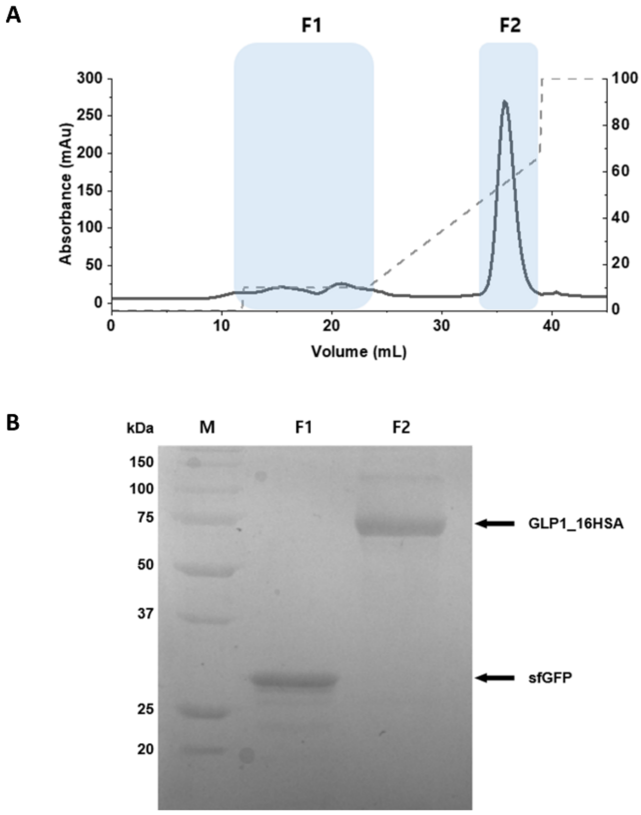

Figure S5. Purification of GLP1_16HSA after proteolytic cleavage by Factor Xa. After the proteolytic cleavage of sfGFP-GLP1_16HSA by Factor Xa, anion exchange chromatography was carried out to purify GLP1_16HSA. (**A**) The anion exchange chromatogram. The cleaved mixture of sfGFP and GLP1_16HSA was desalted with PD-10, loaded onto a HiTrap Q-HP column equilibrated with 20 mM Bis-Tris (pH 6.0), and eluted with an NaCl gradient. The fractions were collected and evaluated on protein gel (B). The dotted line represents the percentage of Buffer B (20 mM Bis-Tris with 1 M NaCl; pH 6.0). (**B**) Protein gel image associated with the anion exchange chromatogram. Protein molecular weight standards are shown in lane M. Cleaved sfGFP was observed in F1. GLP1_16HSA was observed in F2, as indicated in the gel image.

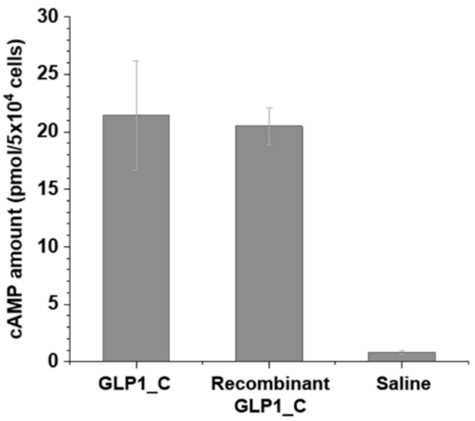

Figure S6. Comparative study of the biological activity of chemically synthesized GLP1_C (GLP1_C) and recombinant GLP1_C in GLP-1R-expressing cells. The cAMP production levels of GLP1_C and the recombinant GLP1_C in the GLP-1R-expressing cells were compared. The recombinant GLP1_C was obtained by processing sfGFP-GLP1_C with 1/500 (*w*/*w*) Factor Xa protease at room temperature for 12 h. There was no further purification. The concentration of both GLP1_C peptides was 10^{-7} M.

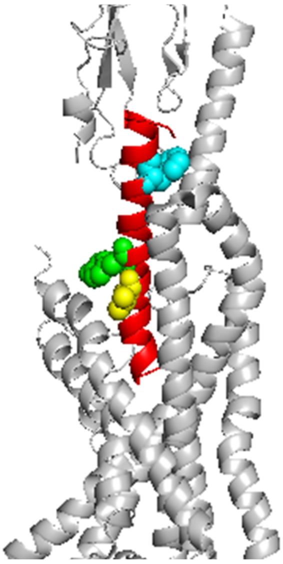

Figure S7. Location of V16, Y19 and F28 residues on Cryo-EM structure of the activated Glucagon-like peptide-1 receptor. The activated GLP-1R with bound GLP-1 is colored in gray, and the bound GLP-1 is colored in red. Each V16 residue, Y19 residue, and F28 residue (PDB ID: 5vai) is marked with yellow, green and cyan respectively. The difference on direction of side chain is observed on the image.
